# Technical challenges and prospects for ex vivo heart perfusion: a republication of the review published in Japanese journal of artificial organs

**DOI:** 10.1007/s10047-026-01550-1

**Published:** 2026-03-21

**Authors:** Daisuke Sakota, Ryo Kosaka, Eiki Nagaoka, Tomoki Tahara, Katsuhiro Ohuchi, Tetsuhito Kigata, Ichiro Sakanoue, Tomoyuki Fujita, Toshihiro Okamoto

**Affiliations:** 1 Health and Medical Research Institute, National Institute of Advanced Industrial Science and Technology (AIST), Tsukuba, Japan; 2 Department of Cardiovascular Surgery, Graduate School of Medical and Dental Sciences, Institute of Science Tokyo, Tokyo, Japan; 3 Department of Clinical Engineering, Faculty of Medical Science, Juntendo University, Urayasu, Japan; 4 Division of Animal Life Science, Graduate School of Agriculture, Tokyo University of Agriculture and Technology, Fuchu, Japan; 5 Department of Thoracic Surgery, Kyoto University, Kyoto, Japan; 6 Department of Thoracic and Cardiovascular Surgery, Cleveland Clinic, Cleveland, USA; 7 Department of Inflammation and Immunology, Lerner Research Institute, Cleveland Clinic, Cleveland, USA; 8 Transplant Center, Cleveland Clinic, Cleveland, USA

**Keywords:** Heart transplantation, Ex vivo heart perfusion, Resting mode, Working mode, Left ventricular assist device

## Abstract

Ex vivo heart perfusion (EVHP) has been clinically utilized for the preservation and functional assessment of donor hearts before heart transplantation. In the field of EVHP research, perfusion methods are often referred to as “modes”. This manuscript describes the characteristics of the EVHP modes that have been proposed to date. The resting mode (Langendorff mode) involves cannulation of the aorta and exclusive coronary perfusion. It is the simplest EVHP mode, and all currently available clinical EVHP systems employ this perfusion method. In contrast, the working mode allows perfusate inflow from the left atrium, thereby generating preload and enabling cardiac output. In our experimental studies, the working mode demonstrated enhanced myocardial metabolism compared with the resting mode; however, it was also associated with increased energy consumption due to cardiac contraction. To address this issue, we developed a left ventricular assist device (LVAD) mode, in which cardiac contraction is supported by a LVAD pump operating in the working mode. LVAD mode was associated with significantly better cardiac function than the resting and working mode. The preservation of cardiac function during EVHP is considered to be greatly influenced by the perfusion mode. Further development of optimized modes will be essential for the effective preservation and evaluation of donor hearts in the future. This review was created based on a translation of the Japanese review written in the Japanese Journal of Artificial Organs in 2024 (Vol. 53, No. 3, pp. 210–215).

## Introduction

Since the United States (U.S.) Food and Drug Administration (FDA) approved TransMedics’ Organ Care System (OCS) Heart in 2021, the U.S. has been actively expanding its donor pool for transplantation, as well as conducting pre-transplant evaluations of “marginal donor hearts” that remain questionable for transplantation. The importance of machine perfusion, or ex vivo heart perfusion (EVHP) or ex situ heart perfusion (ESHP), of explanted donor hearts lies in the preservation and evaluation of the donor heart. The historical evolution of EVHP and the current status of OCS hearts in clinical practice have been described in many references [1–3]. This paper describes the characteristics of EVHP devices from a systems engineering perspective.

## Perfusion “mode” of EVHP

1. Preload and afterload expression methods

In the field of EVHP research, perfusion methods are often referred to as “modes”. The modes of EVHP are more diverse than those of machine perfusion of other organs. Anatomically, the heart is composed of a right heart and a left heart, since there is an inflow (preload) and an outflow (afterload) in each. In EVHP, since the metabolic and mechanical activities of the myocardium are determined by each preload and afterload condition, proper representations of the preload and afterload are important aspects of the device concept.

**Fig. 1:**
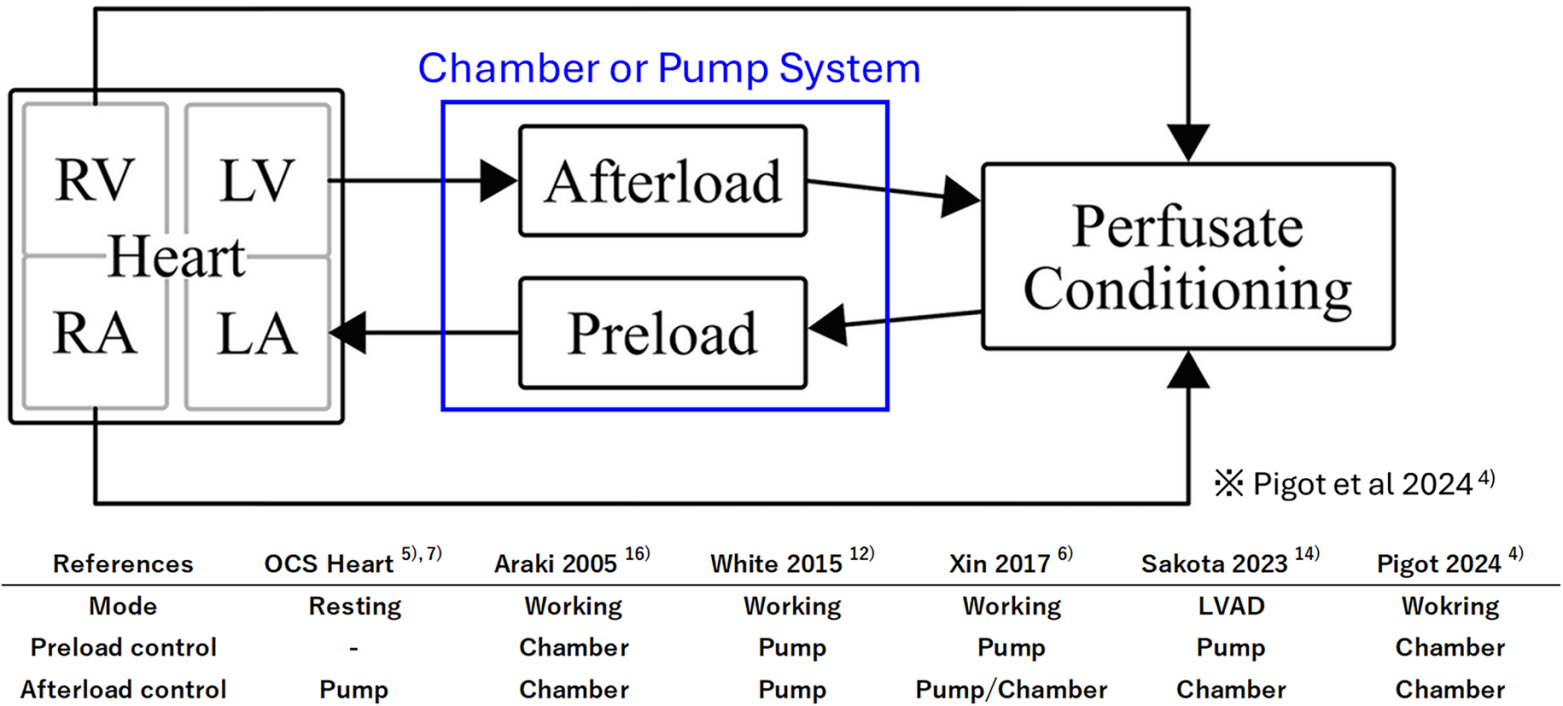
Ex vivo heart perfusion configuration. LA, left atrium; LV, left ventricle; RA right atrium; RV, right ventricle

In vivo, arterial pressure is mainly governed by cardiac output and peripheral vascular resistance. In contrast, in EVHP, the right and left cardiac circuits are separated, and the heart may remain in a state of cardioplegic arrest; thus, an alternative external pressure source is necessary to establish preload and afterload. The pressure source can be categorized into two principal types: chamber-based and pump-driven systems (Fig. 1) [4]. A chamber-based system eliminates the need for complex mechanical control and allows the generation of preload and afterload conditions via hydrostatic pressure determined by the height differential between the heart and the chamber. However, this approach requires a certain reservoir capacity, thereby increasing the priming volume of the EVHP circuit. In addition, sufficient elevation is needed to produce the desired hydrostatic pressure, leading to an increase in the overall system size. In contrast, the pump-based system can be made smaller, but the system structure is more precise and complex because it requires pump control according to cardiac function during perfusion.

2) Resting mode (or Langendorff mode, non-working mode)

In the *OCS Heart User Guide* [5], the perfusion protocol of the device is designated the “Resting mode”. This configuration is also commonly referred to as the Langendorff mode [6], in honor of the German physiologist Oscar Langendorff, who pioneered ex vivo heart perfusion experiments in mammals in the late 19th century. This method applies pressure to the aorta and perfuses only the coronary arteries, which supply the myocardium. Since no perfusate flows into the left atrium, there is no preload, and this approach represents a perfusion mode with afterload only. Because of its simple system configuration, this classical perfusion method has long been used for pharmacological evaluation experiments on the heart.

**Fig. 2:**
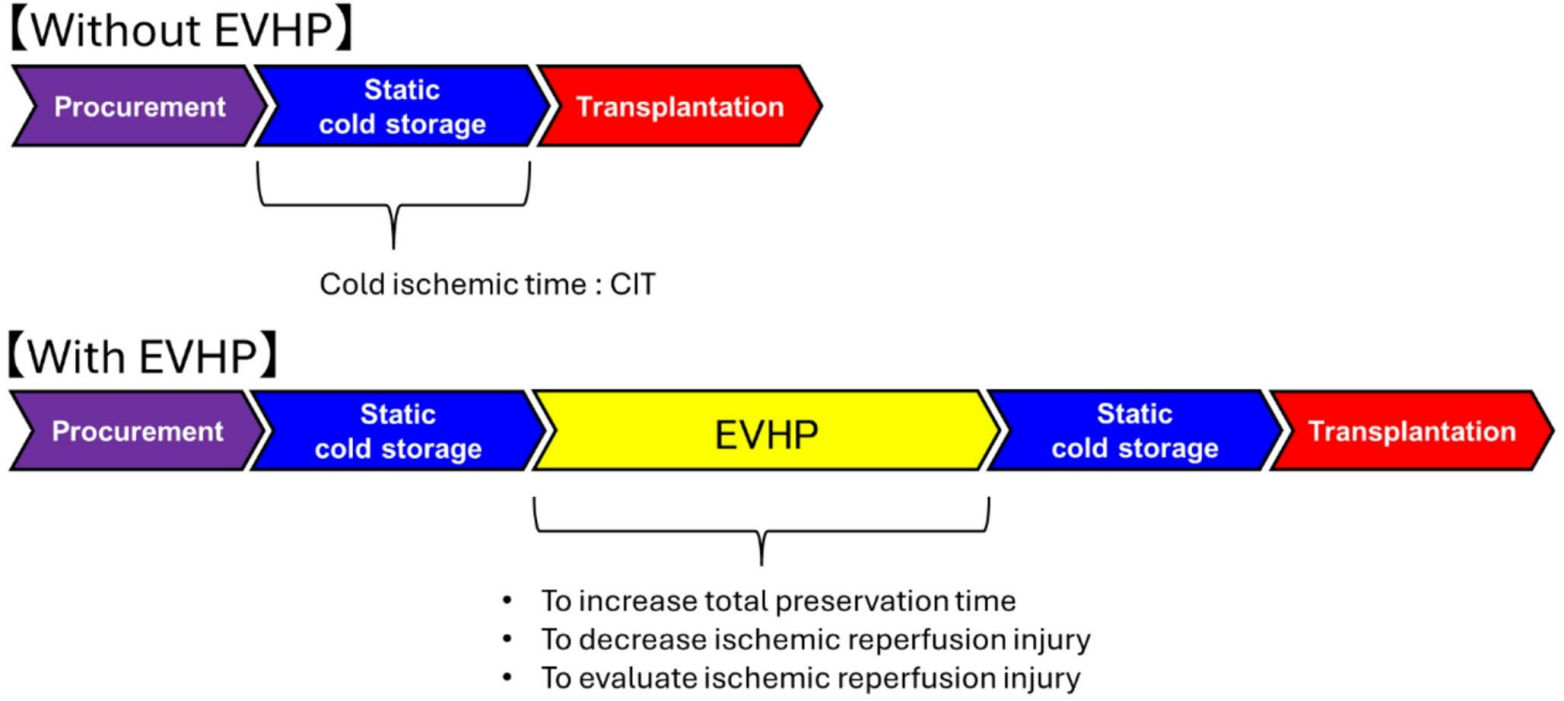
Flow chart showing the progression from donor heart procurement to transplantation

One of the characteristic features of EVHP in the context of heart transplantation is that cold storage is always required both before and after EVHP (Fig. 2). Specifically, because time is required between donor heart procurement and connection to the EVHP system, as well as between the completion of EVHP and the end of transplantation, the heart must remain in a cardioplegic, cold-preserved state during these intervals. Therefore, EVHP must be initiated with the heart in a cardioplegic state, which can be achieved only in the resting mode. Other perfusion modes described later cannot be performed when the heart is arrested; typically, perfusion is initiated in the resting mode and subsequently transitioned to other modes. In addition, when EVHP is terminated, cardioplegic solution is often flushed through the coronary arteries to allow the heart to return to cold storage. Because active cardiac ejection would interfere with this process, it must be performed in the resting mode. Thus, regardless of the specific type of EVHP, it is fundamental that the system be capable of operating in the resting mode at both the initiation and termination of perfusion.

3) Trends in clinical EVHP

To date, all clinically applied EVHP systems have used only the resting mode. In the *Proceed II* trial of *OCS Heart*, the total preservation time in the OCS group was 324 min, with no significant difference in post-transplant outcomes compared with the 195-min cold storage group [7]. In 2023, a multicenter analysis based on *United Network for Organ Sharing* (UNOS) data reported outcomes of heart transplantation following evaluation of donation after circulatory death (DCD) donor hearts using *OCS Heart* [3]. Of 90 controlled DCD cases, defined as having a warm ischemic time of ≤ 30 min with a mean aortic pressure ≤ 50 mmHg or peripheral arterial oxygen saturation ≤ 70%, 80 hearts (89%) underwent transplantation after EVHP with *OCS Heart*. Although the incidence of primary graft dysfunction (PGD) was higher than in brain-dead donor transplants, the 6-month survival rate was comparable to that of the brain-dead group.

XVIVO Inc. is also currently developing a hypothermic system for clinical application. In this resting mode protocol, the system cycles through three phases: coronary perfusion at 20 mmHg for 15 min at 8 °C, coronary perfusion at 10 mmHg for 40 min at 8 °C, and no perfusion for 5 min. In preclinical studies, porcine hearts were preserved for 24 h using this protocol and subsequently transplanted, demonstrating satisfactory post-transplant cardiac function [8]. In the clinical trial phase, six hearts (*n* = 6) underwent transplantation following a total preservation time of 223 min, achieving a 100% survival rate at 180 days post-transplant [9].

In 2024, a case was reported in which a donor heart from the French West Indies was transported to Paris, a distance of 6750 km, via *Air France* and successfully transplanted after a total preservation time of 12 h and 6 min (including approximately 10 h and 32 min of perfusion) [10]. Further reports of successful long-term preservation using this approach are anticipated.

4) Working mode

In contrast to the resting mode, this perfusion method includes preload, allowing the heart to generate cardiac output. Whereas the resting mode can be performed under cardiac arrest, the working mode requires active myocardial contraction and is therefore limited to normothermic EVHP. As described above, when evaluating cardiac function in DCD hearts, it is necessary to identify both hearts suitable for transplantation and those that should be excluded from transplantation. These two objectives inherently involve a trade-off relationship. From a safety standpoint, the latter, excluding unsuitable hearts, should be prioritized, although this results in stricter evaluation criteria. In donor heart assessment using OCS Heart, the standard evaluation parameters are limited to the stability of lactate levels and perfusion parameters [3, 11]. To further expand the donor pool, more precise cardiac functional assessment, particularly mechanical performance evaluation, is required. In cardiac mechanics, there are various parameters that quantitatively describe mechanical cardiac function, for example, cardiac output (L/min), stroke work (mmHg·mL), dP/dt maximum (mmHg/s), dP/dt minimum (mmHg/s), tau (ms), ejection fraction, and others [6, 12–16]. Since these parameters depend on preload conditions, they cannot be adequately evaluated in the resting mode.

Since Neely and Morgan first reported a rat working-mode EVHP in 1967 [2, 17], various improvements have been developed; however, the method remains in the research stage and has not yet been translated into a commercial product. In the working mode developed by Neely and Morgan, both preload and afterload are determined by the height of the fluid chambers. In cardiac physiology, preload is, strictly speaking, defined as the end-diastolic volume (EDV) according to the Frank–Starling law; however, because accurate measurement of EDV is invasive and technically challenging, mean left atrial pressure (LAP) is commonly used as a surrogate parameter and is controlled accordingly in EVHP systems. Considering a setup in which a fixed hydrostatic pressure is applied to the left atrium by adjusting chamber height, an increase in left ventricular ejection fraction (LVEF) would enhance venous return into the left atrium, thereby reducing mean LAP. Conversely, when LVEF is decreased, mean LAP increases. Thus, under constant chamber height, a lower mean LAP indicates better mechanical cardiac performance. Consequently, in EVHP systems using a fixed chamber height to define preload, mean LAP changes continuously in response to cardiac function during working mode perfusion.

Alternatively, a working mode configuration using a centrifugal pump for preload generation, in which a pressure-feedback control system maintains the mean LAP at a predetermined target value, has been reported [6, 12]. In this setup, cardiac output and aortic pressure under constant mean LAP can be used as indices of cardiac performance. Because preload can be kept constant, mechanical cardiac function can be assessed more precisely than with fixed chamber height systems. However, it should be noted that this control system may produce a positive feedback phenomenon: when cardiac function is exceptionally high, the preload pump speed may continue to increase to sustain the target LAP, leading to excessive augmentation of cardiac workload.

Afterload is also a critical factor, because it directly affects coronary perfusion. Similar to the in vivo condition, coronary blood flow increases during diastolic aortic pressure, when the aortic valve is closed, making control of diastolic pressure essential. In the resting mode, since there is no cardiac ejection, diastolic pressure control is relatively straightforward. Furthermore, the flow through the aortic root corresponds to coronary flow, allowing coronary perfusion to be assessed easily (although attention must be paid to the presence of aortic valve insufficiency). In contrast, in the working mode, cardiac output acts as a disturbance to these parameters. To estimate mean coronary flow in working mode, the superior and inferior venae cavae are typically ligated, and the perfusate returning from the coronary circulation is directed into the pulmonary artery, where flow is measured.

Although circuit designs that mimic in vivo aortic pressure, such as those developed for mock circulatory loop studies in artificial heart research, have recently been reported [18], applying such designs to EVHP systems is challenging due to limitations in priming volume and the risk of hemolysis. When a chamber is used to provide afterload, it should be as small and simple as possible to minimize these effects. Historically, the Windkessel chamber method has long been used for adjustment of diastolic pressure in chamber-based systems [19]. In pump-based systems, a working mode configuration has been proposed in which a centrifugal pump is driven in the direction opposite to cardiac output to generate afterload [6, 12]. Although this approach allows for a more compact circuit, it complicates the interpretation of cardiac functional assessment. In a chamber-based system, the chamber functions as a resistive element generating aortic pressure; thus, as long as the chamber parameters remain unchanged, the resistance, and, therefore, aortic pressure, can be regarded as constant. Conversely, when a pump is used, aortic pressure results from a combination of the pressures generated by both the heart and the pump, necessitating caution when interpreting cardiac performance. In such systems, the target pressure for the afterload pump is typically set to the diastolic aortic pressure [12]. However, when cardiac output is low, an increase in pump speed changes afterload and increases the pump-derived pressure component of aortic pressure, causing the measured aortic pressure to no longer accurately reflect cardiac performance. Gellner et al. investigated these effects using a custom-designed working-mode EVHP system with porcine hearts, comparing configurations using a chamber-based afterload (passive afterload working mode: PAWM) and a pump-based afterload (pump-supported working mode: PSWM). Their analysis of cardiac performance during perfusion and post-transplant cardiac function showed that PAWM more closely reflected post-transplant outcomes than PSWM [13].

**Fig. 3:**
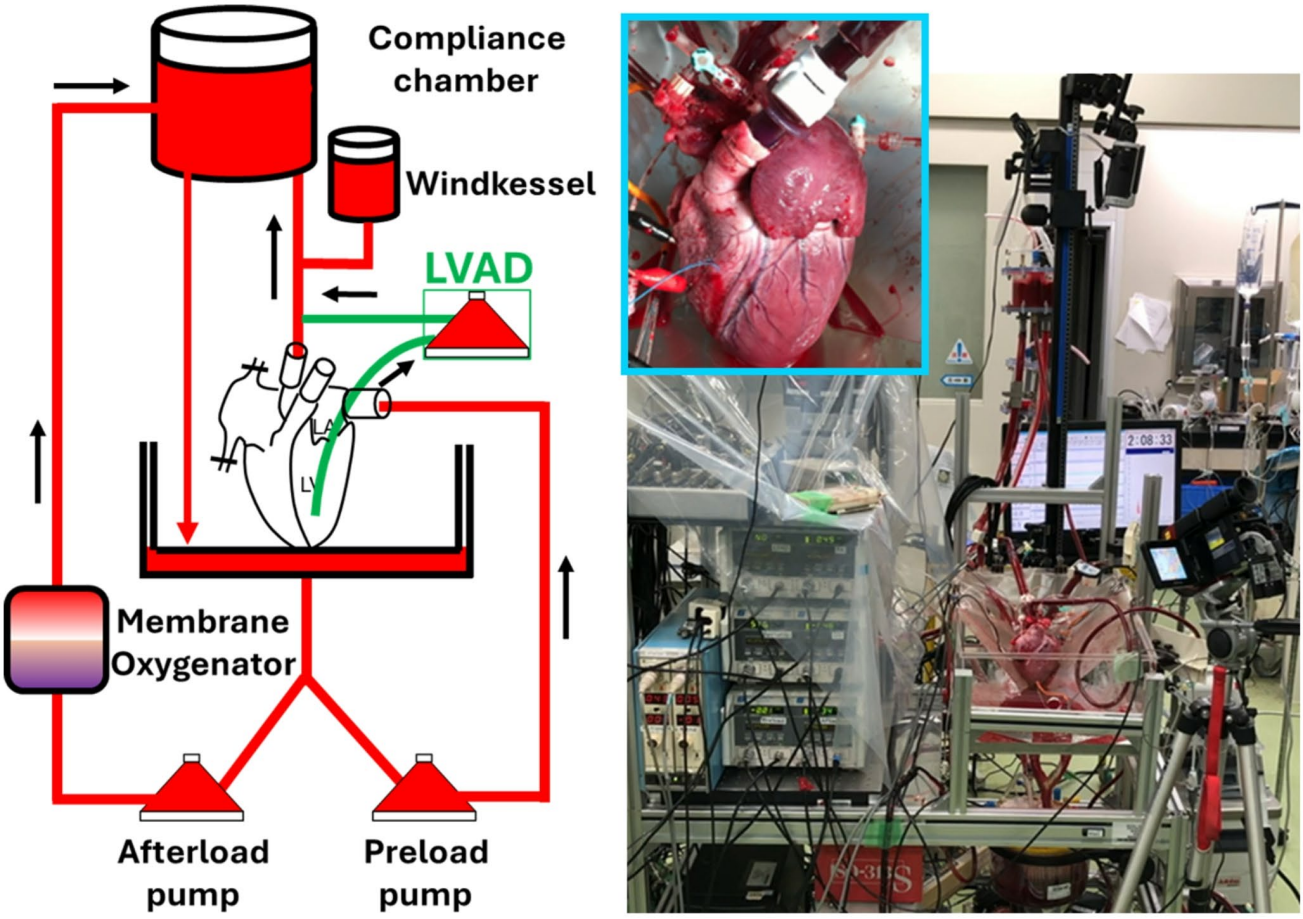
LVAD mode EVHP

5) Left ventricular assist device (LVAD) mode (Fig. 3)

LVAD mode was independently developed by our research team [14]. Although *OCS Heart* has contributed significantly to expanding the donor pool in global heart transplantation, the inability to perform mechanical cardiac function assessment during perfusion remains an unresolved issue. Therefore, practical implementation of the working mode has been eagerly anticipated, and we began developing a proprietary EVHP system based on this concept. However, though the working mode was expected to be useful for functional evaluation, its role in preservation of cardiac function had not been clarified. Because the introduction of preload forces the myocardium to perform mechanical work, it could theoretically increase myocardial energy consumption and potentially induce myocardial fatigue during EVHP. Hatami et al. conducted a 12-h comparative perfusion study in porcine hearts using the PSWM EVHP system they developed and reported that the working mode group showed superior preservation of cardiac function than the resting mode group [15]. However, as mentioned previously, the PSWM configuration inherently causes variation in afterload conditions even within the same group when cardiac function differs, complicating direct comparisons. Because the key distinction between resting and working modes lies in the presence or absence of preload, meaningful comparison requires identical afterload conditions. Using *OCS Heart*, which relies on the resting mode, there is currently no definitive evidence demonstrating a dramatic extension of preservation time compared with conventional cold storage. Reports vary widely, from cases of 16-h preservation [20] to those describing severe edema and primary graft failure after more than 8 h of perfusion [21]. Given this background, the working mode has been globally anticipated as a next-generation EVHP technology. Nevertheless, it inevitably increases system complexity and may potentially be disadvantageous for myocardial preservation. Establishing fundamental knowledge regarding the role of preload in EVHP, namely, the development of working mode systems, is essential for furthering the development of next-generation EVHP platforms. Achieving a balance between functional assessment and myocardial preservation will be a critical requirement for such future systems.

In our EVHP system, a pump-based preload configuration and a chamber-based afterload configuration were adopted. This setup allowed us to maintain constant aortic root resistance and analyze the relationship between the presence or absence of preload and cardiac preservation. In comparative experiments using porcine hearts, the resting mode group showed slightly superior cardiac performance than the working mode group. Moreover, measurements of myocardial adenosine triphosphate (ATP) content after EVHP showed significantly higher levels in the resting mode group. Although the preload and afterload conditions of the working mode have not yet been fully optimized, no marked enhancement in cardiac preservation attributable to preload was observed. However, in the working mode, significant increases were observed in myocardial oxygen consumption, perfusion fluid glucose consumption, and lactate production, indicating that both aerobic and anaerobic glycolytic metabolism were enhanced with preload [14]. Although this may reflect increased ATP synthesis, the concomitant increase in mechanical workload likely resulted in greater ATP consumption, which ultimately did not contribute to improved preservation of myocardial function.

Since perfusate inflow from the atrium to the ventricle occurs by passive filling, myocardial energy consumption is primarily associated with systolic contraction. Simply reducing preload, however, would effectively convert the system into the resting mode. This led us to consider the potential utility of selective systolic unloading. Our newly developed perfusion method, termed the LVAD mode, can be formally described as a co-pulse, heartbeat-synchronized, left ventricular assist-type, working mode. This approach aims to induce left ventricular filling (i.e., increased EDV) via preload, while simultaneously unloading the ventricle at the onset of systole using a centrifugal pump functioning analogously to an LVAD. Consequently, diastole mimics a working mode state, whereas systole approximates a resting mode state. Although complete synchronization and total ventricular drainage cannot be achieved in practice, an assist ratio of approximately 71% and a synchronization accuracy of about 76% within 100 ms were achieved. In the LVAD mode group, stroke work preservation after 6 h of EVHP reached approximately 75%, compared with 30% in the resting mode group and 31% in the working mode group [14].

## Summary and future prospects

In this paper, we described the system configurations of the resting mode, the working mode, and the LVAD mode newly developed by our group. Normothermic ex vivo heart perfusion (EVHP) serves two principal purposes: organ preservation and functional assessment. Regarding the capability for cardiac preservation among these three modes, it remains unclear whether the resting mode or the working mode is superior [1]. Under our experimental conditions, preservation outcomes in the resting mode were superior to those in the working mode; however, further investigation is required to determine which mode is more advantageous for preservation [14]. Notably, the LVAD mode demonstrated approximately twice the stroke work preservation rate compared with the other two modes. Regarding the capability for cardiac functional assessment, the resting mode does not allow evaluation of mechanical cardiac function, whereas such assessment is possible in the working mode. However, most working modes reported to date are designed primarily for mechanical functional assessment of the left heart. Regarding evaluation of the right heart, the development of bi-ventricular working mode systems has also been reported; however, such systems are even more complex [22, 23]. The LVAD mode can function as a conventional working mode when the assist ratio is set to 0%, thereby allowing cardiac functional assessment. Whether cardiac function can be reliably assessed while myocardial contraction is actively assisted by the LVAD pump has not yet been investigated.

From the perspective of clinical application, it is desirable for a system to provide superior preservation and functional assessment capabilities; however, simplicity of the device and safe operability are also critically important. The resting mode has the simplest system configuration, but its limitation lies in the difficulty of assessing mechanical cardiac function. In contrast, the working mode and the LVAD mode are associated with increased system complexity. In addition, because cardiac output is generated, the perfusate flow rate increases compared with resting mode, and hemolysis is likely to occur. Since excessive hemolysis increases the concentration of potassium ions in the perfusate and can affect cardiac function, the longer the perfusion time, the more hemolysis becomes a problem. In addition, software development has been insufficient. Robust control of pre- and post-loading according to cardiac function is essential for safe protection of the heart during the working mode and for proper assessment of cardiac function. All of this represents a mechanical structure problem, which can only be solved by mechanical engineering. Unlike other organs, the heart is an autonomously moving organ, and its role is a mechanical function of pumping, so the participation of mechanical engineers is desirable. The author has been involved in the development of artificial hearts for many years, and the LVAD mode was inspired by his research on artificial hearts. We hope that this paper will inspire artificial organ engineers to become involved in the development of next-generation EVHP systems.
